# Role of Point-of-Care C-reactive Protein Testing on Antibiotic Prescription in Febrile Children: A Randomized Controlled Trial

**DOI:** 10.7759/cureus.92646

**Published:** 2025-09-18

**Authors:** Simran Khanna, Mohammad Haseeb, Akul Pania, Vadde Y Reddy, Madhurasree Nelanuthala, Avinash L Sangle

**Affiliations:** 1 Paediatrics, Mahatma Gandhi Mission (MGM) Medical College and Hospital, Aurangabad, IND; 2 Pediatrics and Neonatology, Mahatma Gandhi Mission (MGM) Medical College and Hospital, Aurangabad, IND; 3 Pediatrics, Mahatma Gandhi Mission (MGM) Medical College and Hospital, Aurangabad, IND; 4 Pediarics, Mahatma Gandhi Mission (MGM) Medical College and Hospital, Aurangabad, IND

**Keywords:** antibiotics prescription, antimicrobial resistance, crp: c-reactive protein, febrile illness, point-of-care testing (poct)

## Abstract

Background

Overprescription of antibiotics in febrile children contributes significantly to antimicrobial resistance. Point-of-care testing (POCT) for C-reactive protein (CRP) may help differentiate bacterial from viral infections and support more rational antibiotic use.

Objective

To assess the effectiveness of POCT CRP testing in reducing antibiotic prescription rates among febrile children in an outpatient pediatric setting.

Methods

A randomized controlled trial was conducted at a tertiary care hospital in Maharashtra, India, involving 208 children aged one month to 18 years with febrile illness (≤5 days). Participants were randomized into two groups: Group 1 (n=106) received POCT CRP testing, and Group 2 (n=102) received standard clinical care. CRP was measured using the LumiraDx fluorescence immunoassay (LumiraDx UK Ltd, Alloa, UK). The primary outcome was the rate of antibiotic prescription.

Results

Antibiotic prescription was significantly higher in Group 1 (59.4%) than in Group 2 (46.1%) (p=0.023). Within the POCT group, 84.2% of children with CRP >20 mg/L were prescribed antibiotics compared to 45.6% with CRP ≤20 mg/L (p=0.0001). Elevated CRP levels were significantly associated with antibiotic prescription, especially in cases of fever and abdominal pain.

Conclusion

POCT CRP influenced physicians' prescribing decisions, particularly when CRP levels exceeded 20 mg/L. However, without structured integration into clinical algorithms, its use alone did not reduce overall antibiotic use. Physician training and adherence to CRP-based guidance are essential to optimize the benefits of POCT CRP in antimicrobial stewardship.

## Introduction

Antibiotic overuse in children with febrile illnesses remains a key driver of antimicrobial resistance, particularly in low- and middle-income countries [1]. Most febrile illnesses in pediatric practice are viral and self-limiting, but diagnostic uncertainty often leads to the empirical use of antibiotics [2]. Apart from diagnostic uncertainty, other factors contributing to the overuse of antibiotics are concerns about missing serious bacterial infections; in resource-limited settings, physicians might also be concerned about patients' perceived or actual inability to access health care if their condition deteriorates. Precise and prompt distinction between bacterial and viral illnesses is crucial to ensure suitable antibiotic use and reduce the spread of antibiotic resistance. Rapid tools to assist in decision-making are needed to reduce this over-reliance on empirical therapy. Biomarkers are increasingly valued for diagnosing bacterial infections. An effective biomarker would help physicians quickly and accurately identify bacterial infections in patients with acute febrile illnesses. C-reactive protein (CRP), an acute-phase protein made by the liver during infection, could serve as such a biomarker. It is commonly elevated in bacterial infections and can aid in differentiating these from viral etiologies [3,4].

Point-of-care testing (POCT) for CRP provides rapid results, allowing for evidence-based prescribing at the time of consultation. C-reactive protein point-of-care testing reduced antibiotic use for non-severe acute respiratory tract infections without compromising patients' recovery in primary health care in Vietnam [5]. The acceptability and impact of POCT CRP testing in children in India, where antimicrobial resistance is a growing concern, remain underexplored. This study addresses this gap by evaluating the role of POCT CRP in guiding antibiotic prescriptions in children with acute febrile illness in a pediatric outpatient setting in India, where unrestricted antimicrobial access and varying clinical practices may influence outcomes.

## Materials and methods

Trial design

This study was a prospective, open-label, randomized controlled trial with a parallel group design, conducted to compare the effect of POCT CRP testing versus standard clinical care on antibiotic prescription rates in febrile children. No changes were made to the trial design after commencement.

Trial setting

The trial was conducted in the outpatient pediatric department of Mahatma Gandhi Mission (MGM) Medical College and Hospital, a tertiary care facility in Chhatrapati Sambhajinagar (Aurangabad), Maharashtra, India, serving a diverse urban and rural population with high rates of antimicrobial resistance due to widespread antibiotic access.

Eligibility criteria

Eligible participants were children aged one month to 18 years presenting with an acute febrile illness (temperature ≥38°C for ≤5 days). Inclusion criteria included outpatient status and informed consent from parents or guardians (and assent from children aged ≥7 years). Exclusion criteria encompassed severe illness requiring hospitalization (e.g., sepsis, severe dehydration), trauma, acute respiratory distress, known chronic inflammatory conditions (e.g., autoimmune diseases), or use of antibiotics within the previous 7 days.

Intervention and comparator

Participants were randomized into two groups. Group 1 (POCT CRP group, n=106) underwent CRP testing using the LumiraDx CRP Test platform (LumiraDx, Martel Instruments Ltd, Stanley, UK) before clinical consultation. The test used a finger-prick capillary blood sample processed via a microfluidic fluorescence immunoassay, providing CRP values within four minutes. A threshold of 20 mg/L was used to guide interpretation, with values >20 mg/L suggesting a higher likelihood of bacterial infection, as suggested in the study by Muller et al. [5]. Clinicians were advised to integrate CRP results with clinical judgment but were not mandated to follow a strict protocol. Group 2 (control group, n=102) received standard clinical evaluation and treatment based on history, physical examination without CRP testing. Both groups were managed by the same team of pediatricians to ensure consistency in clinical decision-making.

Outcomes

The primary objective of this study was to evaluate the antibiotic prescription rate in febrile children with and without POCT CRP testing. The secondary objectives included assessing the distribution of CRP values among children in the POCT CRP group, examining the correlation between CRP levels and clinical features, and analyzing prescribing patterns across different diagnostic groups. No changes were made to the outcome measures during the trial.

Sample size

Based on a previous study reporting antibiotic prescription rates of approximately 64% in standard care and a potential reduction to 44% with POCT CRP testing [6], a sample size of 208 children (104 per group) was calculated to achieve 80% power with an alpha of 0.05, using a two-sided chi-square test. This calculation assumed no loss to follow-up, as the primary outcome was assessed at the point of consultation. No interim analyses were planned or conducted.

Allocation concealment mechanism

Randomization was performed using a computer-generated random number sequence with a 1:1 allocation ratio, prepared by an independent statistician not involved in patient management. Allocation was concealed using sequentially numbered, opaque, sealed envelopes opened only at the time of participant enrollment to prevent selection bias.

Statistical methods

Data were analyzed using SPSS v24.0 (IBM Corp., Armonk, NY, USA). The primary outcome, antibiotic prescription rate, was compared between groups using a chi-square test. Secondary outcomes, including the distribution of CRP values and prescribing patterns by diagnosis, were analyzed using descriptive statistics and chi-square tests. Continuous variables, such as age and CRP levels, were compared using independent t-tests. All analyses were conducted on an intention-to-treat basis. A p-value <0.05 was considered statistically significant. Missing data were minimal (<2% for primary outcome) and handled using listwise deletion, as they were deemed missing completely at random (e.g., due to incomplete documentation). No subgroup or adjusted analyses were performed due to the limited sample size.

Recruitment and follow-up

Participants were recruited from May 2023 to November 2024, during regular outpatient clinic hours. Follow-up for outcomes was limited to the initial consultation, as the primary outcome (antibiotic prescription) was assessed at the point of care. No long-term follow-up was conducted for benefits (e.g., recovery rates) or harms (e.g., adverse events, re-visits), as the study focused on immediate prescribing decisions.

Ethics

The study was approved by the MGM-Ethics Committee for Research on Human Subjects (ECRHS) on April 27, 2023 (approval number: MGM-ECRCH/2023/40). It was registered with Clinical Trials Registry India (Trial REF/2024/07/088719, date 13/12/2023). Written informed consent was obtained from parents or guardians of all participants, and assent was obtained from children aged ≥7 years. The committee reviewed and approved the study protocol, informed consent form, assent form, case record form, principal investigator’s curriculum vitae, and undertaking. The trial adhered to the Declaration of Helsinki and local ethical guidelines. No study team members participated in the ethics committee’s decision-making process.

## Results

**Figure 1:**
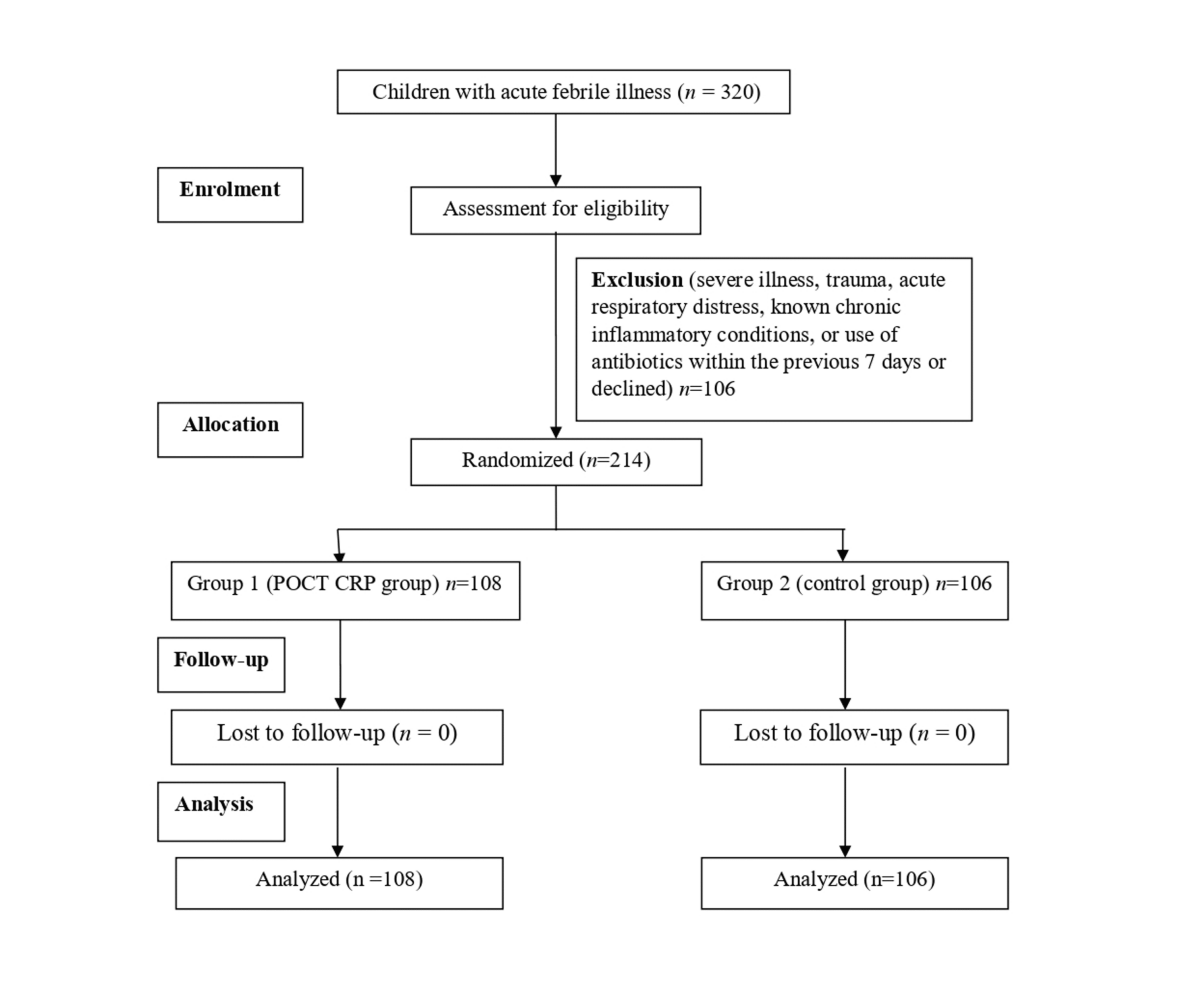
CONSORT Flow Diagram Depicting the Study Participants

A total of 320 children with acute febrile illness were screened, of whom 214 met the eligibility criteria and were enrolled and randomly assigned between April 27, 2023, and April 26, 2025. In Group 1 (POCT CRP), 106 children were assigned, received the intended intervention (CRP testing via the LumiraDx platform), and were analyzed for the primary outcome (antibiotic prescription rate). In Group 2 (control), 102 children were assigned, received standard clinical care without CRP testing, and were analyzed for the primary outcome. No participants were lost to follow-up, as the primary outcome was assessed at the point of consultation. The flow of participants through the trial is depicted in Figure 1.

Baseline characteristics were comparable between groups. The mean age of children in Group 1 was 92.34 ± 63.21 months (range: 3-204 months), while in Group 2 it was 89.44 ± 72.49 months (range: 2-408 months), with no statistically significant difference (t=0.31, p=0.756). The sex distribution was similar, with males constituting 47.2% in Group 1 and 53.9% in Group 2 (χ²=1.20, p=0.273). The most common provisional diagnosis in both groups was acute respiratory infection (ARI), reported in 46.2% of children in Group 1 and 42.1% in Group 2 (χ²=0.40, p=0.527). Acute febrile illness (AFI) accounted for 41.5% in Group 1 and 38.3% in Group 2 (χ²=0.13, p=0.717). Acute gastroenteritis (AGE) was observed in 12.3% of children in Group 1 and 19.6% in Group 2 (χ²=1.35, p=0.244). One case of meningitis was diagnosed in Group 2. These differences in diagnosis distribution were not statistically significant (Table 1).

**Table 1:** Baseline Characteristics and Antibiotic Prescription Rates in Two Groups N=number, SD=standard deviation. P-values for age were calculated using an independent t-test; p-values for sex, diagnosis, and antibiotic prescription were calculated using chi-square tests. The test statistic column reports the t-value for age and chi-square (χ²) values for categorical variables. AGE: acute gastroenteritis, ARI: acute respiratory infection, AFI: acute febrile illness.

Variable	Group 1 (POCT CRP)	Group 2 (Control)	Test Statistic	P-value
Age Mean (SD)	92.34 (63.21)	89.44 (72.49)	t = 0.31	0.756
Sex N (%)				
Male	50 (47.2)	55 (53.9)	χ² = 1.20	0.273
Female	56 (52.8)	47 (46.1)		
Diagnosis N (%)				
AGE	13 (12.26)	20 (19.6)	χ² = 1.35	0.244
ARI	49 (46.22)	43 (42.15)	χ² = 0.40	0.527
AFI	44 (41.50)	37 (38.27)	χ² = 0.13	0.717
Meningitis	0 (0.0)	1 (1.0)	-	-
Antibiotic Prescribed N (%)				
Yes	63 (59.4)	47 (46.1)	χ² = 5.17	0.023
No	43 (40.6)	55 (53.9)		

Antibiotic prescription rates differed significantly between the two groups. In the POCT CRP group (Group 1), 63 out of 106 children (59.4%) received antibiotics, compared to 47 out of 102 children (46.1%) in the standard care group (Group 2), with a statistically significant difference (χ²=5.17, p=0.023) (Table 1).

Among children in Group 1, CRP values varied widely. The majority (67.0%) had CRP levels ≤20 mg/L, while 15.1% had levels between 21 and 40 mg/L. Smaller proportions had CRP levels between 41-60 mg/L (6.6%), 61-80 mg/L (2.8%), and 81-100 mg/L (1.9%). Only 5.7% of children had CRP values exceeding 120 mg/L. When stratified by CRP level, antibiotic prescription was significantly higher in children with CRP >20 mg/L, where 32 out of 38 children (84.2%) received antibiotics, compared to 31 out of 68 children (45.6%) with CRP ≤20 mg/L (χ²=15.02, p=0.0001). This indicates a strong association between elevated CRP levels and antibiotic use (Table 2).

**Table 2:** Antibiotic Prescription Rates by POCT CRP Levels in Group 1 P-values were calculated using a chi-square test. The test statistic column reports the chi-square (χ²) value.

POCT CRP Value	Antibiotic Prescribed	Antibiotic Not Prescribed	Total	Test Statistic	P-value
≤20 mg/L	31 (45.5%)	37 (54.4%)	68	χ² = 15.02	0.0001
>20 mg/L	32 (84.2%)	6 (15.7%)	38		
Total	63	43	106		

Further subgroup analysis by clinical diagnosis and CRP levels showed a trend toward increased antibiotic use in children with ARI and AFI when CRP was elevated, although these did not reach statistical significance. In ARI cases, 88.2% of children with CRP >20 mg/L received antibiotics compared to 64.5% with CRP ≤20 mg/L (χ²=3.13, p=0.077). A similar pattern was seen in AFI, where 88.2% of children with high CRP received antibiotics versus 64.5% with lower CRP levels (χ²=2.73, p=0.099). For AGE and meningitis, there was no statistically significant difference in antibiotic use based on CRP values (χ²=0.41, p=0.522 for AGE; χ²=0.32, p=0.571 for meningitis) (Table 3).

**Table 3:** Association Between Diagnosis, POCT CRP Values, and Antibiotic Prescription P-values were calculated using chi-square tests. The test statistic column reports the chi-square (χ²) value. AGE: acute gastroenteritis, ARI: acute respiratory infection, AFI: acute febrile illness.

Diagnosis	POCT CRP Value	Antibiotics Prescribed: Yes	Antibiotics Prescribed: No	Test Statistic	P-value
AGE	≤20 mg/L	3	8	χ² = 0.41	0.522
	>20 mg/L	1	1		
ARI	≤20 mg/L	20	11	χ² = 3.13	0.077
	>20 mg/L	15	2		
AFI	≤20 mg/L	19	11	χ² = 2.73	0.099
	>20 mg/L	15	2		
Meningitis	≤20 mg/L	3	0	χ² = 0.32	0.571
	>20 mg/L	3	1		

Safety data

No systematic collection of harms or unintended events was conducted, as the study focused on immediate antibiotic prescribing decisions at the point of consultation. No adverse events related to the POCT CRP testing procedure (e.g., finger-prick complications) were reported in Group 1. In both groups, no immediate harms, such as allergic reactions to prescribed antibiotics or procedural complications, were documented during the consultation. However, the lack of long-term follow-up precludes assessment of delayed harms, such as antibiotic-related adverse effects or treatment failures.

## Discussion

This randomized controlled trial investigated the impact of point-of-care C-reactive protein (POCT CRP) testing on antibiotic prescription rates in febrile children within a resource-limited pediatric outpatient setting in India. Contrary to expectations, the intervention did not lead to a reduction in overall antibiotic use; instead, the POCT CRP group exhibited a higher antibiotic prescription rate (59.4%) compared to the control group (46.1%, p = 0.023). This finding provides nuanced insights into the role of POCT CRP in clinical decision-making, revealing both its potential and the challenges associated with its implementation in a setting with high antimicrobial resistance and variable prescribing practices. The increased antibiotic prescription rate in the POCT CRP group likely stems from clinicians’ reliance on elevated CRP levels (>20 mg/L) to guide treatment decisions, as evidenced by 84.2% of children with elevated CRP receiving antibiotics compared to 45.6% with CRP ≤20 mg/L (p = 0.0001). Physicians often associate elevated C-reactive protein (CRP) levels with bacterial infections and may initiate antibiotic therapy based on this parameter. However, CRP is a non-specific acute-phase reactant that can also be elevated in viral infections and various non-infectious inflammatory conditions, such as autoimmune diseases and tissue injury* *[3,4]. Studies have highlighted that sole reliance on CRP values may lead to inappropriate antibiotic prescribing if not combined with clinical judgment and guidelines. CRP levels alone may not capture the full clinical picture, potentially leading to over- or under-prescription of antibiotics [7,8]. Other factors that might have influenced antibiotic prescription are, possibly, diagnostic uncertainty, parental expectations, or entrenched prescribing habits prevalent in resource-limited settings [9]. In low- and middle-income countries like India, where unrestricted access to antibiotics and socioeconomic factors influence prescribing practices, such challenges are particularly pronounced [10].

The effectiveness of POCT CRP hinges on its integration into a broader framework of clinical practice. Combining CRP POCT with other interventions, such as antimicrobial stewardship programs, delayed prescribing strategies, and enhanced communication skills, can effectively reduce antibiotic overuse [11,12]. Physician training is equally critical, as the lack of standardized education on CRP interpretation in this trial may have led to inconsistent application of test results [13]. Additionally, in settings where parental demand for antibiotics is high, POCT CRP can serve as an objective tool to justify withholding antibiotics when CRP levels are low, provided clinicians are equipped to communicate these findings effectively to caregivers [14]. Addressing these factors is essential to maximizing the benefits of POCT CRP in antimicrobial stewardship.

Comparisons with existing literature reveal mixed outcomes for POCT CRP. A study by Do et al. in Vietnam reported a 14% reduction in antibiotic use with POCT CRP, attributed to structured implementation and physician training [5]. Conversely, Van den Bruel et al. found no significant reduction in antibiotic prescribing among children unless general practitioners fully embraced the diagnostic value of CRP [15]. Our findings align more closely with the latter, underscoring the importance of implementation strategies. A systematic review by Petel et al. highlighted that biomarkers like CRP are most effective when paired with clinical decision-making tools tailored to local healthcare contexts [16]. Similarly, Keitel et al. demonstrated in Tanzania that POCT CRP reduced antibiotic prescribing when integrated with electronic decision-support tools, emphasizing the need for structured protocols [17]. These studies collectively suggest that the success of POCT CRP depends on its integration into a supportive clinical framework.

Strengths and limitations

This study represents the first randomized controlled trial to evaluate the LumiraDx CRP POCT platform in an Indian pediatric outpatient setting, offering valuable insights into its real-world feasibility and influence on antibiotic prescribing in a resource-limited context with high antimicrobial resistance. The balanced baseline characteristics of the two groups and the clear definition of outcomes enhance the validity of the findings. However, several limitations must be acknowledged. The open-label design may have introduced bias, as clinicians were aware of group assignments, potentially influencing prescribing decisions. The lack of long-term follow-up for outcomes such as re-visits or complications limits the ability to assess the broader impact of POCT CRP on patient safety and recovery. Additionally, the absence of a standardized protocol for integrating CRP results into clinical decision-making likely contributed to variability in prescribing practices. The single-center design and regional prescribing habits may limit the generalizability of the findings to other settings. Finally, the relatively small sample size restricted the power for subgroup analyses, potentially missing nuanced differences in prescribing patterns across diagnostic groups.

Future directions

To maximize the benefits of POCT CRP, future research should focus on larger multicenter trials across diverse healthcare settings to assess generalizability. Developing and validating CRP-integrated clinical decision algorithms tailored to low- and middle-income countries is critical. Educational interventions to standardize CRP interpretation and address physician and caregiver concerns are also needed. Additionally, evaluating long-term outcomes, including revisit rates, complications, and antimicrobial resistance trends, will provide a more comprehensive understanding of POCT CRP’s impact. Cost-effectiveness analyses are essential to assess the feasibility of scaling POCT CRP in resource-limited settings. Finally, exploring the combination of POCT CRP with other biomarkers, such as procalcitonin, or rapid viral diagnostics could enhance diagnostic accuracy [18].

## Conclusions

The use of point-of-care CRP testing in febrile children has the potential to guide more rational antibiotic prescribing when integrated with clinical assessment. While in this study, the availability of CRP values led to increased antibiotic use overall, it also helped identify children less likely to benefit from antibiotics when CRP was low. Thus, it highlighted the need for structured implementation and clinician training to maximize its utility. Given the small sample size and single-center study, our findings should be interpreted with caution. Future large-scale, multicenter trials are warranted to establish standardized CRP thresholds and evaluate their role within diagnostic algorithms in pediatric outpatient and emergency settings.
